# Small Changes in Environmental Parameters Lead to Alterations in Antibiotic Resistance, Cell Morphology and Membrane Fatty Acid Composition in *Staphylococcus lugdunensis*

**DOI:** 10.1371/journal.pone.0092296

**Published:** 2014-04-08

**Authors:** Marcus J. Crompton, R. Hugh Dunstan, Margaret M. Macdonald, Johan Gottfries, Christof von Eiff, Timothy K. Roberts

**Affiliations:** 1 Department of Chemistry, Gothenburg University, Göteborg, Sweden; 2 Institute of Medical Microbiology, University of Münster, Münster, Germany; 3 Metabolic Research Group, School of Environmental and Life Sciences, The University of Newcastle, Callaghan, Australia; State Key Laboratory of Pathogen and Biosecurity, Beijing Institute of Microbiology and Epidemiology, China

## Abstract

*Staphylococcus lugdunensis* has emerged as a major cause of community-acquired and nosocomial infections. This bacterium can rapidly adapt to changing environmental conditions to survive and capitalize on opportunities to colonize and infect through wound surfaces. It was proposed that *S. lugdunensis* would have underlying alterations in metabolic homeostasis to provide the necessary levels of adaptive protection. The aims of this project were to examine the impacts of subtle variations in environmental conditions on growth characteristics, cell size and membrane fatty acid composition in *S. lugdunensis*. Liquid broth cultures of *S. lugdunensis* were grown under varying combinations of pH (6–8), temperature (35–39°C) and osmotic pressure (0–5% sodium chloride w/w) to reflect potential ranges of conditions encountered during transition from skin surfaces to invasion of wound sites. The cells were harvested at the mid-exponential phase of growth and assessed for antibiotic minimal inhibitory concentration (MIC), generation time, formation of small colony variants, cell size (by scanning electron microscopy) and membrane fatty acid composition. Stress regimes with elevated NaCl concentrations resulted in significantly higher antibiotic resistance (MIC) and three of the combinations with 5% NaCl had increased generation times (P<0.05). It was found that all ten experimental growth regimes, including the control and centroid cultures, yielded significantly different profiles of plasma membrane fatty acid composition (P<0.0001). Alterations in cell size (P<0.01) were also observed under the range of conditions with the most substantial reduction occurring when cells were grown at 39°C, pH 8 (514±52 nm, mean ± Standard Deviation) compared with cells grown under control conditions at 37°C with pH 7 (702±76 nm, P<0.01). It was concluded that *S. lugdunensis* responded to slight changes in environmental conditions by altering plasma membrane fatty acid composition, growth rates and morphology to achieve optimal adaptations for survival in changing environments.

## Introduction

*Staphylococcus lugdunensis* is a member of the coagulase negative group of staphylococci and was first described in 1988 [1]. Historically, coagulase negative staphylococci (CoNS) have been considered as innocuous skin commensals and their presence in pathological samples has often been dismissed as contamination [2], [3]. Their roles as pathogenic staphylococci have only recently been acknowledged and the infections they cause have often tended to be sub-acute or chronic displaying a smaller array of tissue-damaging toxins and enzymes compared to the more virulent *Staphylococcus aureus* [4]. *S. lugdunensis* exists as part of the natural human skin flora and is frequently found colonizing the perineal region. *S. lugdunensis* commonly causes typical CoNS wound infections, indwelling foreign device infections and endocarditis [5] but can also cause aggressive infections with a more fulminant course which resemble infections caused by *S. aureus*. Furthermore, *S. lugdunensis* is frequently misidentified as *S. aureus* because of their similar colony appearance when cultured on blood agar with yellow to gold pigmentation displaying extensive hemolysis. Some isolates of *S. lugdunensis* can also produce a membrane-bound coagulase [1].

Bacteria are known for their ability to adapt to rapidly changing environmental conditions [6]. In stressful or fluctuating environments, the formation of small colony variants (SCVs) or colonies showing increased biofilm secretion can confer a survival advantage [1]. This versatility has allowed them to survive in harsh dynamic environments with cells capable of rapid phenotype-switching even though they are all genetically identical cells [7]. When the environment returns to favorable conditions, the bacteria can switch back to the “wild-type” phenotype [8].

Survival in a range of external environments on fomites between human hosts demands the ability to adapt to broad ranges of continuously changing environmental conditions involving temperature, pH, nutrient and water availability as well as exposure to toxic chemicals. Once colonized on the skin surface as a commensal, the environmental parameters of temperature, pH, osmotic pressure and water availability would be more stable. In the event of a wound breaking the skin surface, the bacterium must go through a rapid process of transition into an opportunistic pathogen. To achieve infection of the damaged tissues, the bacterium would need to switch on virulence factors and rapidly adjust to altering environmental conditions to optimize metabolism and avoid host defense systems for successful invasion. Thus, when colonizing human host surfaces and transitioning for invasion of a wound site, the bacteria must be able to rapidly adapt to localized changes in pH, temperature and osmotic pressure. The human wound site varies in regard to tissue type and the ongoing processes associated with the specific phases of healing and recovery. The pH of a chronic wound site can vary from 7.5–8.9 and as the wound progresses towards healing, the pH in the site changes towards neutral and then becomes acidic [9]. It has been shown that sodium concentrations increase in wound sites immediately after traumatization with a concomitant drop in potassium and magnesium. The skin temperature can also vary depending on the body location and the external conditions, averaging 2–4°C less than the core temperature [10]. Localized inflammation and healing processes can result in elevations (+2–4°C) in the cutaneous wound temperature [11]. These rapid response features which facilitate infection, surface colonization and transfer between hosts imply that the cells react to environmental stimuli by altering their metabolism. Changes in metabolic rate, protein synthesis, enzyme function, structural adaptations and alterations in membrane composition may be involved in the response to environmental challenge. Epigenetic alterations in phenotype to optimize opportunistic proliferation and survival may be a principal factor contributing to pathogenicity.

The specific variations in temperature, pH and osmotic pressure represent relatively subtle variations in environmental conditions and little is known about the metabolomic responses by staphylococci to these low level environmental changes or their combinations. A recent investigation using FTIR provided evidence that variable sets of environmental conditions resulted in distinct phenotypes of *S aureus* each with characteristic FTIR profiles [12]. The plasma membrane has been considered essential for the survival of microorganisms since these structures represent the interface between the cytoplasm and the external environment [13]. Membrane lipids have been associated with diverse roles including respiration, metabolism, protein transport, synthesis of the peptidoglycan wall and the initiation of chromosome replication and cell division [14], [15], [16] and [17]. The integrity and maintenance of function of the plasma membrane would be vital to the cell's survival and it has been proposed that altering membrane fatty acid composition and fluidity would provide potential mechanisms for adaptation to the changing environment [18].

*S. lugdunensis* is a commensal bacterium emerging as an important nosocomial infectious agent with demonstrated capacities to rapidly take advantage of wound-sites for opportunistic infections. This current study focused on testing the hypothesis that subtle changes in environmental conditions would lead to significant alterations in cell membrane composition, antibiotic resistance to Gentamicin, generation time, and ultrastructural characteristics in *S. lugdunensis*. The study design was established to investigate the potential for identifying contiguous epigenetic responses to varying combinations of environmental conditions designed to mimic potential ranges observed in wound sites.

## Materials and Methods

### Bacterial Samples

A clinical isolate of *S. lugdunensis* derived from an earlier investigation collecting coagulase negative isolates from patients suffering from chronic muscle pain [19] was further investigated for responses to environmental stresses [20]. The isolate had been maintained as culture stock in the laboratory on Columbia Base Agar with 5% Horse Blood (HBA) (Oxoid). The isolate had been appropriately stored and routinely sub-cultured to maintain viability. Identity checks were routinely performed using API Staph^R^ Biochemistry and through PCR of the 16SrRNA gene using the method of Brown *et al.*, [21] and the results verified through the NCBI BLAST database.

### Bacterial growth and harvest

*S. lugdunensis* cultures were grown in Brain and Heart Infusion broth (BHI, Oxoid Ltd. Basingstoke) with a flask volume to culture volume of 5∶1 at 37°C in an orbital shaking incubator at 120 rpm. Broths that required modification (pH and solute concentration) were adjusted with 1M Hydrochloric acid (HCl), 1M Sodium hydroxide (NaOH) or HPLC grade NaCl prior to autoclave sterilization. Gentamicin (sulfate salt) was filter-sterilized through 0.22 µm membrane filters (Millex^R^ -GP, PES, 0.22 µm) prior to addition to sterile broth. Following growth of broth cultures under the various experimental conditions, the cells were inoculated onto Columbia Base Agar with 5% Horse Blood (HBA) (Oxoid) at 37°C to determine the relative abundances of SCV colonies. The colonies were characterized as SCVs if they presented as pinpoint colonies <1 mm in diameter with reduced haemolytic activity and pigmentation at 24–28 hrs post incubation (10), (24). The larger colonies were classified as wild type (WT). The SCVs were then sub-cultured onto HBA plates under optimal conditions at 37°C to demonstrate reversion and their identities were checked using API Staph (Biomerieux).

Experimental cultures were harvested at the mid-exponential phase of growth (∼3 hours) as determined by monitoring culture absorbance (Abs_600 nm_). The bacterial cells were harvested by centrifugation (6000× g) for 20 minutes and subsequently washed 3 times (6000× g, 20 minutes) with MilliQ water to remove confounding contributions from the growth media. The washed cells were re-suspended in 1 ml of MilliQ water whereupon aliquots were aseptically removed to assess ultrastructure by Scanning Electron Microscopy (SEM) and antibiotic susceptibility. The remaining cell suspension was subsequently snap frozen with liquid nitrogen and allowed to thaw. This freeze-thawing process was repeated an additional 3 times to ensure adequate cell lysis. The lysed cells were then once again frozen and then lyophilized using a FTS Systems Freeze Dryer (Flexi-Dry MP).

### Susceptibility to gentamicin

Following growth under defined environmental stresses the susceptibility to gentamicin was determined using a modification of the procedure described by Wallace *et al.* [22]. A stock solution of BHI containing 4 mg/mL gentamicin was serially diluted with fresh BHI to provide final antibiotic concentrations of 2 mg/mL, 1 mg/mL, 500 µg/mL, 250 µg/mL, 125 µg/mL, 62.5 µg/mL, 31.3 µg/mL, 15.6 µg/mL, 7.8 µg/mL, 3.9 µg/mL and 1.9 µg/mL in separate wells of a 96 well ELISA plate. Each sample was plated out in triplicate and 35 µL of the washed bacterial cell suspension was then added to each of the wells. The plates were incubated for 16 hours at 37°C with 5% CO_2_. The MIC was determined by aseptically plating out a 3 µL aliquot from each well onto Mueller Hinton Agar (MHA) plates and bacterial cell growth assessed after 16 hours of incubation with 5% CO_2_ at 37°C.

### Microscopy

To prepare the SEM slides, a 3 µl sterile metal loop was inserted into the final washed bacterial suspension and smeared onto a 12 mm cover glass slip. After air-drying in a sterile environment, cells were stained for 1 minute with filtered crystal violet, rinsed in sterile water and decolorized with 95% ethanol. Samples were stored in a desiccator before sputter coating with 20 nm of gold and viewed under a Scanning Electron Microscope (Philips XL-30 Model + Oxford ISIS EDS) at 15 kv at magnifications of 10,000–40,000. Cell diameter measurements were recorded using the UTHSCSA (University of Texas Health Science Centre at San Antonio) Image Tool, version 3, available as a free download from http//ddsdx.uthscsa.edu/dig/itdesc.html. With the exception of treatment 4 (pH 8, 35°C, 5% added NaCl w/w) which had only five to six cells *per* field of view, at least 10–20 cells were measured *per* SEM frame from a total of 141 to 185 cells *per* sample.

### Membrane fatty acid analyses

The membrane fatty acid analysis was carried out using direct transesterification through the method of Lepage and Roy [23] with the following modifications: a solution of methanol (MeOH) and toluene in a 4∶1 (v/v) ratio which contained 19.2 µg per mL of C23∶0 (Tricosanoic acid) as an internal standard were prepared. Samples of lyophilized and lysed *S. lugdunensis* cells were placed into pre-weighed 8 ml borosilicate glass screw-top tubes and the masses of harvested bacterial cells were determined. A volume of 2 mL of 4∶1 MeOH/toluene (4∶1v/v) solution with internal standard was then aliquoted directly onto the dried cell masses. The samples were placed on ice before addition of 200 µL of acetyl chloride (Sigma Aldrich). The samples were then double sealed with Teflon and vortexed at a moderate speed (5–10 seconds), and heated at 100°C for 1 hour to form fatty acid methyl esters (FAMEs). Cooled samples were neutralized through the addition of 5 ml of 6% potassium carbonate solution (K_2_CO_3_). To separate the phases clearly, the samples were centrifuged for 10 minutes at 3,000 rpm, with the upper toluene phase transferred immediately to a 400 µL glass insert held within a 2 mL GC autosampler vial. The lower methanol/water phase and protein interface were discarded. The samples were analyzed immediately through Gas Chromatography – Mass Spectrometry (GC-MS) and surplus stored at −25°C under desiccation.

All GC-MS analyses were conducted on a Hewlett-Packard 6890 series gas chromatograph coupled with a Hewlett-Packard 5973 mass selective detector. Separation of the FAMEs was achieved through a SGE BPX5 column, 30 m in length with internal dimensions of 0.25 mm with a 0.25 µm film thickness. The carrier gas was Helium (He). Injection of the sample was through an Agilent 7673 injector with the following settings: injection volume 1 µl; splitless injection; He as the carrier gas with a flow rate of 0.5 ml/min; electron voltage 70 eV. The GC oven had a starting temperature of 80°C, held for 3 minutes, ramping to 300°C at 3°C per minute, with a final hold time of 10 minutes at 300°C for a total run time of 85.3 minutes. Both the GC inlet and the heated transfer line were held at 280°C.The fatty acids were identified on the basis of retention time characteristics as well as mass spectral data with analytical standards for confirmation (Supelco 37 component FAME mix). Standards of iso- and anteiso-Hexadecanoic acid (iC17∶0 and aiC17∶0, Sigma Aldrich) were also used for elucidation of the position of the methyl branch on the FAMEs in relation to the retention time.

### Experimental design

**Figure 1:**
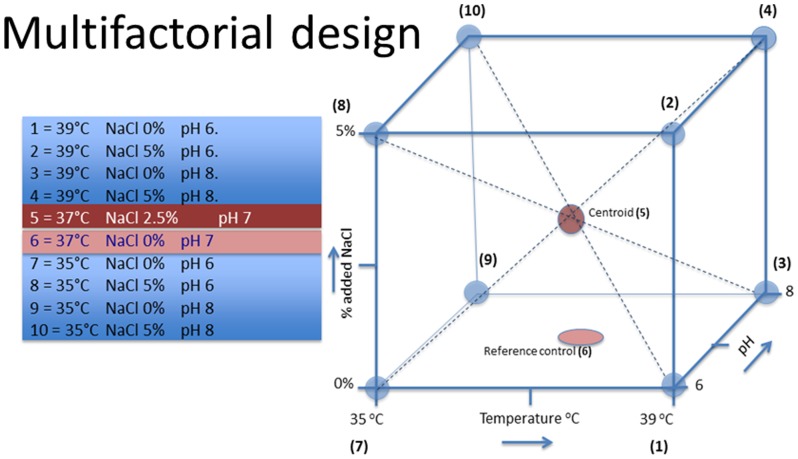
The experimental design matrix showing the variation of three environmental parameters to investigate the synergistic impacts of small alterations of pH, temperature and osmotic pressure on *S. lugdunensis*.

Broth cultures of *S. lugdunensis* were exposed to a range of environmental conditions by varying temperature (35°C, 37°C and 39°C), pH (6.0, 7.0 and 8.0) and osmotic pressure (0%, 2.5% and 5% added NaCl w/w) according to factorial experimental design. Combinations of environmental conditions were selected to represent the potential ranges of the three environmental parameters in wound sites as summarized in Figure 1. The reference control samples were incubated at 37°C and pH 7, with no added NaCl and were represented on the lowest horizontal plane in the cube design. The “centroid” (37°C, pH 7 and 2.5% added NaCl) sample represents the mid-point of the variable parameters and was essential to enable evaluation of the continuum response of *S. lugdunensis* to the changes in the growth conditions.

The experimental design resulted in 9 sets of combinations of pH, temperature and osmotic pressure along with a reference control, where *S. lugdunensis* was grown in BHI until the mid-exponential phase of growth and the times taken to reach this predetermined cell density were recorded. Due to the logistical constraints, the incubations were performed over a 5-day period. The reference control and centroid sets (sample sets 6 and 5 respectively in Figure 1) were performed in triplicate on days 1, 3 and 5 yielding a total of 9 replicates for each of these sample sets. The remaining 8 combinations of environmental conditions (representing sample sets 1, 2, 3, 4, 5, 8, 9, 10 in Figure 1) each had 3 replicate sample cultures. The 35°C incubations were performed on day 2 and the 39°C incubations were performed on day 4. This allowed for assessing variance in the control samples across the experimental period for the reference control and centroid samples and allowed an evaluation of within treatment reproducibility. Following growth under the 10 combinations of environmental conditions, each set of replicate samples were assessed for MIC, SCV formation, generation time, cell size and membrane fatty acid composition as end-point measures.

### Statistics

The data were presented as the mean ± SD of replicate results. UTHSCSA Image Tool, (Version 3) was used to measure cell diameters. The factorial design for this experiment was generated using MODDE software version 9.0 and the data were evaluated by Multiple Linear Regression (MLR), as implemented in the software. Significance tests of MLR coefficients were estimated by cross-validation using jack-knifing within the software. Furthermore, the data were analysed by Analysis of Variance (ANOVA), Tukeys and discriminant function analysis using Statistica (Version 6.1, Statsoft, Tulsa, OK). Multi-dimensional analyses were undertaken by the SIMCA-P+, v.12 using Orthogonal Partial Least Squares (OPLS) [24] with fatty acid data in the descriptive matrix (X) and Gentamicin MIC, Generation time, and SCV formation as independent vectors (Y). Again significance testing for model complexity, *i.e.* number of Orthogonal Partial Least Squares (OPLS) components, was achieved by cross-validation.

## Results

Cultures of *S. lugdunensis* were grown under the prescribed range of environmental conditions with varying combinations of temperature (35–39°C), pH (6–8) and added NaCl (0–5%) resulting in 10 treatments as summarized in Figure 1 and Table 1. The reference control and centroid samples were repeatedly assessed throughout the experimental period to account for potential within group variations throughout the experimental period. Significant differences in MIC, generation time, cell sizes and the formation of SCV phenotypes were noted across all the treatments in comparison with the reference control and centroid samples.

**Table 1:** Summary of endpoint measures for cultures of *S. lugdunensis* following growth under each of the 10 environmental regimes as defined in the experimental design (Figure 1).

Environmental Regime				End point Measures (±SD)			
Code (*n*)	Temp °C	pH	NaCl %	MIC (µg/ml Gentamicin)	Gen time (hours)	Cell size (nm)	SCV %
1*(n = 3)*	35	6	0	4.6^a^	1.14±0.01^a^^,^ c	568±53^a^	0.6±0.3
2*(n = 3)*	35	6	5	62.5^b^	1.23±0.02^a^	613±52^c^^,^^g^	2.1±1.0^a^
3*(n = 3)*	35	8	0	3.9^a^	1.15±0.05^a^^,^ c	582±53^a^^,^ b	0.9±0.5
4*(n = 3)*	35	8	5	41.7^c^	1.22±0.03^a^	727±78^d^	0.8±0.8
***5(n = 9) Centroid***	***37***	***7***	***2.5***	***24.3^d^***	***1.09±0.02*** a *^,^* b *^,^* c	***685±70^e^***	***0.2*** **±0.3**
***6(n = 9) Control***	***37***	***7***	***0***	***6.5*** a	***1.05*** **±** ***0.02*** b *^,^* c	***702±76^e,f^***	***0.4*** **±0.5**
7*(n = 3)*	39	6	0	9.1^a^^,^^d^	0.92±0.03^b^^,^ c	596±60^b^^,^ c	0.1±0.1
8*(n = 3)*	39	6	5	62.5^b^	1.19±0.04^a^^,^ c	634±60^c^^,^^g^	0.7±1.2
9*(n = 3)*	39	8	0	3.9^a^^,^^e^	0.97±0.01^b^^,^ c	514±52^h^	0.5±0.9
10*(n = 3)*	39	8	5	52.1^b^^,^ c	1.05±0.04^b^^,^ c	715±76^d,f^	0.2±0.3

Significant differences were marked by grouping superscripts.

a–c (P<0.05) – *ie* those values with the same superscript are not significantly different.

**Figure 2:**
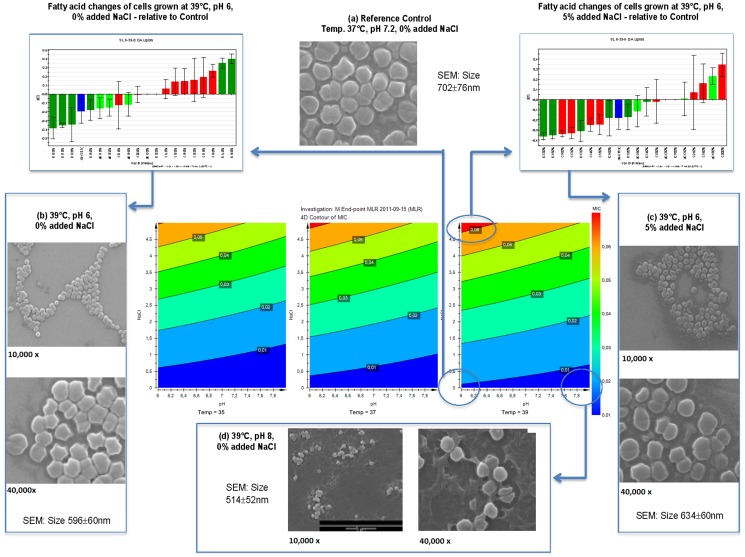
Integrated summary of the cellular responses to the changes in combinations of environmental parameters including temperature, osmotic pressure and pH. The central three coloured contour plots show the influences of the combined stresses of NaCl and pH on the MIC for Gentamicin. The coloured contour plot on the left represents the effect of pH and NaCl concentration on the MIC at 35°C. pH had little effect on changing MIC, whereas higher concentrations of NaCl increased MIC as shown by the change in colour from blue to orange/red. Elevating the temperature as shown in the central plot to 37°C, and then to the plot on the right to 39°C, resulted in some enhanced responses of MIC as NaCl concentrations were increased. The fatty acid profiles measured for the cells grown at 39°C, pH 6 and 5% salt, which generated the highest MIC (circled), displayed substantial differences when compared with the corresponding profiles of the control cells shown in the difference chart on the top right-hand side of the figure. SEM evaluations for these same cells (far right (c)) revealed smaller cell sizes compared with the reference control cells (a). The fatty acid profiles measured for the cells grown at 39°C, pH 6 and 0% salt, which generated low MIC responses (circled), displayed a different pattern of alterations when compared with the corresponding profiles of the control cells shown in the difference chart on the top left-hand side of the figure. SEM evaluations for these same cells with 0% salt (far left (b)) revealed smaller cell sizes compared with the reference control cells (a). At 39°C, cells incubated at pH 8 and 0% NaCl produced the lowest MIC (circled) and had the smallest sized cells of any treatments in the study (d).

The centroid samples represented the mid-point of all of the variable ranges and differed from the defined control regime by having 2.5% w/w added NaCl as the only changed parameter. This addition of the 2.5% w/w NaCl resulted in a significant increase in antibiotic resistance compared with the control samples (Table 1). At the temperatures of 35°C and 39°C at pH 8 and pH 6, the MIC values for 5% w/w added NaCl were significantly higher than both control and centroid samples. The combinations of lower pH and high NaCl concentrations led to the highest antibiotic resistance for gentamicin as shown in the contour plots in Figure 2. Changing temperature and pH alone in these ranges did not significantly alter the antibiotic resistance (Table 1). The addition of 5% NaCl at 35°C and pH 6 or pH 8 resulted in longer generation times compared with the reference control but not compared with the centroid samples (P<0.05). These conditions at pH 6 yielded a higher proportion of SCV colonies following growth (P<0.05, Table 1).

The cells were also subjected to morphological investigations following growth under the various sets of conditions. The SEM analyses involved measures of at least 10–20 cells *per* SEM frame from a total of 141 to 185 cells *per* sample. The reference control cells had an average diameter of 702±76 nm (±SD) as shown in Figure 2(a) and this was not significantly different to either the centroid sample (685±70 nm) or the sample grown at pH 8 and 39°C with 5% added NaCl (715±76 nm). Growth at pH 8 and 35°C with 5% added NaCl resulted in cell diameters (727±78 nm) which were larger than the control samples (P<0.05). Growth under all remaining environmental regimes resulted in significant reductions in cell size compared with the reference control and there were numerous significant differences between the various treatment regimens as shown in Table 1 (P<0.05). The cells grown at pH 8 and 39°C and 0% added NaCl displayed the most notable reduction in cell size (514±52 nm) compared with the control and consistently showed more extracellular matrix material as demonstrated in Figure 2(d).

**Figure 3:**
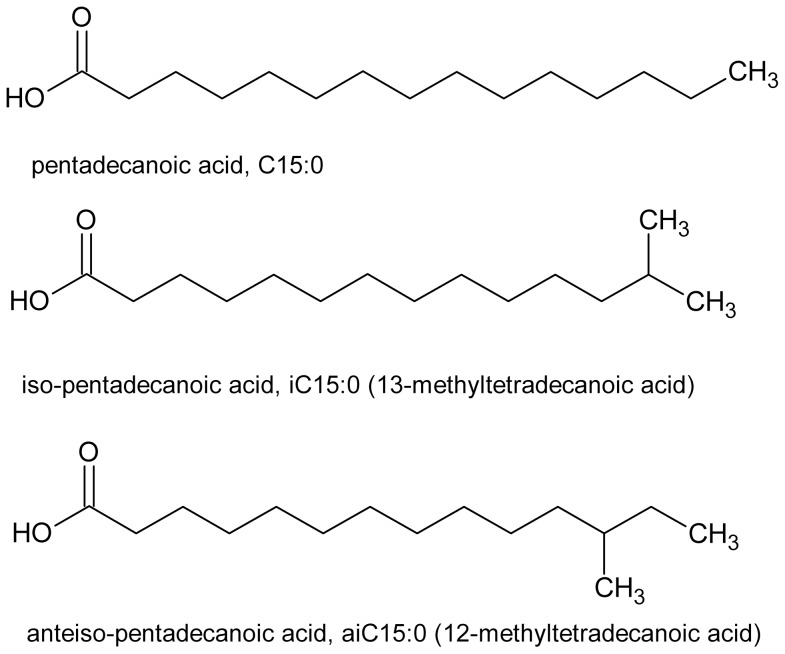
Illustration of the structural differences in C15∶0, iC15∶0 and aiC15∶0 fatty acids representing the minor, mid-range and major components of the cell membrane fatty acids, each of which respond independently to environmental stresses.

The membrane fatty acid compositions of the bacteria were determined for cells following growth under all experimental growth regimes. The total average fatty acid yield for cells grown under the control conditions was approximately 28.6 µg/mg dry weight representing 2.86% of the dry weight mass. The control group was assessed for fatty acid composition on days 1, 3 and 5 to account for variance across the experimental period. The fatty acid composition of staphylococci was characterized by the presence of odd-chain and branched-chain fatty acids as demonstrated for C15∶0, iC15∶0 and aiC15∶0 fatty acids in Figure 3, representing the minor, mid-range and major components of the cell membrane fatty acids.

The analyses revealed that 3 fatty acids displayed substantial variance in concentrations over the sampling days (SD>15% mean); these were aiC15∶0 (8.83±1.4 µg/mg dry weight), iC17∶0 (0.087±0.017 µg/mg dry weight) and aiC17∶0 (0.25±0.083 µg/mg dry weight). The membrane fatty acid profiles were assessed as relative percentage abundances from the eight treatment regimens for comparison with the reference control and centroid samples as summarized in Table 2. Significant differences (P<0.05) were marked with a superscript “^a^” for those significantly different compared with the reference control and “^b^” for differences compared with the centroid sample. These data indicated that the eight stress regimes resulted in significant differences in the relative abundances of the plasma membrane fatty acids compared with the control and centroid reference cultures. Variations were noticed in the total percentages of iso- fatty acid components when comparing the responses with either the reference control (24.4%) or centroid samples (23.3%) with values ranging from 20.9% to 26.9% (P<0.05, Table 2).

**Table 2:** The percentage relative-abundance fatty acid profiles for the reference control and centroid cultures compared with the fatty acid profiles generated in each stress regime during the multi-stress experiment.

Fatty acid % Relative Abundance	Experimental Series ID Code									
	1	2	3	4	5	6	7	8	9	10
	35°C	35°C,	35°C,	35°C,	37°C	37°C	39°C,	39°C,	39°C,	39°C,
	0% NaCl, pH 6	0% NaC pH 8	5% NaCl pH 6	5% NaCl pH 8	2.5% NaCl pH 7 Centroid	0% NaCl pH 7 Control	0% NaCl pH 6	0% NaCl pH 8	5% NaCl pH 6	5% NaCl pH 8
iC13∶0	1.53^ab^±0.08	1.63^ab^±0.10	1.06±0.01	1.75^ab^±0.21	**0.98^a^ 0.11**	**1.13±0.06**	0.9^a^±0.14	1.0^a^±0.09	0.71^ab^±0.06	0.87^a^±0.13
aiC13∶0	0.37^ab^±0.05	0±0.00	0.23^ab^±0.07	0.32^ab^±0.11	**0±0.00**	**0±0.00**	0±0.00	0±0.00	0±0.00	0±0.00
iC14∶0	12.6^ab^±0.44	10.19±0.08	12.5^b^±0.29	11.08±0.14	**10.06±1.25**	**10.83±1.30**	14.7^ab^±0.23	9.33±0.04	10.77±0.23	9.08±0.48
C14∶0	9.67±0.25	9.29±0.14	7.61^a^±0.17	6.79^ab^±0.04	**8.78±1.72**	**9.58±1.17**	9.9±0.25	7.17^a^±0.35	7.64^a^±0.11	6.59^a^±0.46
iC15∶0	10.87±0.08	12.1^ab^±0.09	8.63^ab^±0.07	12.4^a^±0.22	**10.73±0.32**	**10.97±0.54**	8.74^ab^±0.11	10.84±0.72	8.2^ab^±0.09	10.93±0.67
aiC15∶0	36.42±0.26	39.84±0.85	41.62±0.47	38.59±1.43	**40.71±3.91**	**38.53±3.58**	35.81±0.78	45.5^a^±1.34	43.8^a^±1.01	45.9^a^±2.20
C15∶0	1.32^b^±0.04	1.1±0.09	0.85^a^±0.08	1.04±0.16	**1.0^a^±0.23**	**1.37±0.30**	1.57^b^±0.11	0.91^a^±0.10	0.87^a^±0.07	0.5^ab^±0.25
iC16∶0	1.07±0.02	0.67^ab^±0.01	0.84^ab^±0.01	0.95^a^±0.06	**1.02±0.07**	**1.06±0.06**	1.45^ab^±0.02	0.74^ab^±0.07	1±0.04	0.78^ab^±0.08
C16∶0	4.88^b^±0.11	4.76±0.00	3.9^a^±0.10	4.08^a^±0.06	**4.22^a^±0.41**	**4.58±0.24**	4.97^ab^±0.08	4.08^a^±0.03	3.6^ab^±0.03	3.89^a^±0.09
iC17∶0	0.33±0.03	0.3^a^±0.03	0.17^ab^±0.00	0.55^a^±0.06	**0.44±0.11**	**0.38±0.06**	0.22^ab^±0.01	0.39±0.04	0.22^ab^±0.01	0.52^a^±0.03
aiC17∶0	0.92±0.04	0.8±0.03	0.78±0.02	1.26±0.07	**1.33±0.50**	**1.09±0.32**	0.84±0.01	0.94±0.05	1.09±0.08	1.32±0.08
C17∶0	0.76^b^ 0.02	0.68^a^±0.01	0.57^ab^±0.02	0.72±0.11	**0.67^a^±0.04**	**0.83±0.06**	0.9^b^±0.06	0.71^a^±0.09	0.58^ab^±0.01	0.59^ab^±0.04
iC18∶0	0.04^a^±0.03	0±0.00	0±0.00	0.1^a^±0.03	**0.04±0.05**	**0±0.00**	0±0.00	0±0.00	0±0.00	0±0.00
cis-C18∶1(9)	0±0.00	0±0.00	0±0.00	0.11±0.13	**0.1±0.09**	**0.11±0.12**	0±0.00	0±0.00	0±0.00	0±0.00
C18∶0	11.5^a^±0.14	11.55±0.01	11.64±0.36	11.5^a^±0.13	**11.94±0.45**	**12±0.28**	12.26±0.33	11.74±0.35	11.97±0.28	11.67±0.13
iC19∶0	0±0.00	0±0.00	0±0.00	0.09^a^±0.02	**0.04±0.04**	**0.02±0.03**	0±0.00	0±0.00	0±0.00	0±0.00
aiC19∶0	0±0.00	0±0.00	0±0.00	0.07^a^±0.02	**0.03±0.05**	**0.01±0.03**	0±0.00	0±0.00	0±0.00	0±0.00
C19∶0	1.7±0.08	1.56^ab^±0.04	1.89±0.08	1.94±0.26	**1.74±0.11**	**1.87±0.17**	1.98^b^±0.07	1.8±0.20	2.03^b^±0.19	1.7±0.07
C20:	5.93±0.18	5.52±0.28	7.71^ab^±0.37	6.67^a^±0.43	**6.14±0.48**	**5.61±0.31**	5.6±0.14	5.31^b^±0.33	7.46^ab^±0.55	5.69±0.05
C21∶0	0.03±0.00	0^a^±0.00	0.04^a^±0.01	0.05^ab^±0.02	**0.02±0.02**	**0.02±0.01**	0.01±0.01	0^a^±0.00	0.02±0.02	0^a^±0.00
iso FA	26.46^b^±0.47	24.9±0.30	23.16±0.33	26.88^ab^±0.03	**23.31±1.25**	**24.39±1.70**	26.02^b^±0.46	22.3±0.86	20.9^ab^±0.15	22.17±1.35
anteiso FA	37.71±0.29	40.63±0.83	42.63±0.38	40.24±1.25	**42.08±4.44**	**39.63±3.90**	36.66±0.77	46.4^a^±1.30	44.9^a^±0.93	47.19^a^±2.14
Unsaturated	0±0.00	0±0.00	0±0.00	0.11±0.13	**0.1±0.09**	**0.11±0.12**	0±0.00	0±0.00	0±0.00	0±0.00
SCFA	35.81±0.74	34.46±0.53	34.22±0.70	32.8^a^±1.10	**34.51±3.21**	**35.85±2.24**	37.2±0.38	31.72^a^±1.25	34.17±1.06	30.6^a^±0.79

Individual fatty acids were observed to have characteristic response patterns associated with the eight environmental regimes with differing profiles of membrane fatty acids. Certain fatty acids only appeared in the lower temperature regimes and in the control and centroid treatments. These included aiC13∶0, iC18∶0, iC19∶0 and aiC19∶0. The fatty acid aiC17∶0 was the only fatty acid which did not vary its relative abundance in response to the stress regimes in comparison to both the control and the centroid samples. aiC15∶0 was the most abundant fatty acid in the membrane extract under all growth conditions and was primarily influenced by higher temperatures except in culture series 7 (39°C, 0% NaCl, pH 6) where there was no apparent increase in relative abundance compared with the control (Table 2). The monounsaturated fatty acid (MUFA) cis-C18∶1(9) was only detected in three treatments including the control, centroid and series 4 conditions.

The changing of one or two environmental parameters at a lower temperature seemed to have no effect on the % composition of the anteiso- fatty acids which contrasted with the higher temperature regimes where there were significant increases in the relative abundances of anteiso- fatty acids compared with the reference control. Significant reductions in straight chain fatty acids (SCFA) were observed at 35°C (series 4, Table 2) as well as at 39°C (series 8 and 10) compared with the control (series 6).

**Figure 4:**
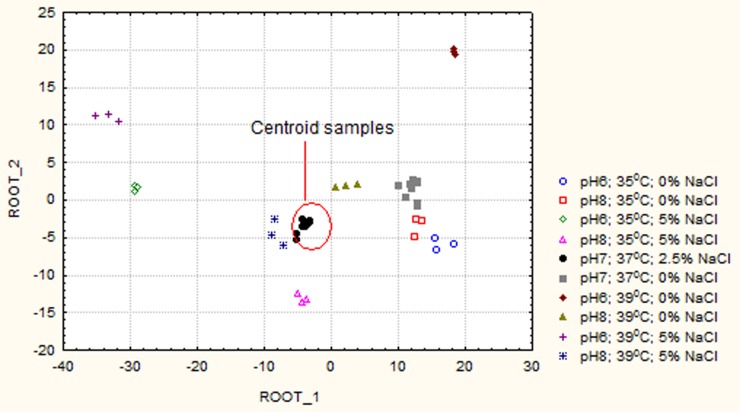
The fatty acid profiles for each treatment sample were essentially reduced to a position on a 2 dimensional scatterplot using multivariate discriminant function analysis. The x-axis represents the discriminant function from the fatty acid data that gave the greatest possible discriminating power between the groups (root-1). The y-axis represents a second discriminant function which has less discriminating power but is orthogonal to the first function by using different aspects of the fatty acid profile (root-2). Effectively, the fatty acid profile data determine the position of the sample on the 2-dimensional plot. Samples with similar fatty acid profiles will thus be positioned next to each other, and those that have different profiles should be separated in space. It is thus clear that samples from each treatment group were clustered together because they have similar fatty acid profiles and each group was well resolved from the other indicating characteristic fatty acid profiles for each combination of environmental factors.

To evaluate the fatty acid profile differences between each of the treatments, the data were assessed by discriminant function analysis. This procedure determined that the membrane fatty acid profiles were significantly different (Wilk's Lambda = 0.0000001, F14.1, P<0.001), with aiC13∶0, iC16∶0, iC17∶0 and aiC15∶0 identified as major contributing factors to the resolution of the profiles. The discriminant function was then used to generate a canonical scatter-plot of all the replicates for each treatment and is shown in Figure 4. This plot clearly shows that all replicate membrane fatty acid profiles were tightly grouped and distinct from each other representing unique adaptations to the various treatment regimes.

**Figure 5:**
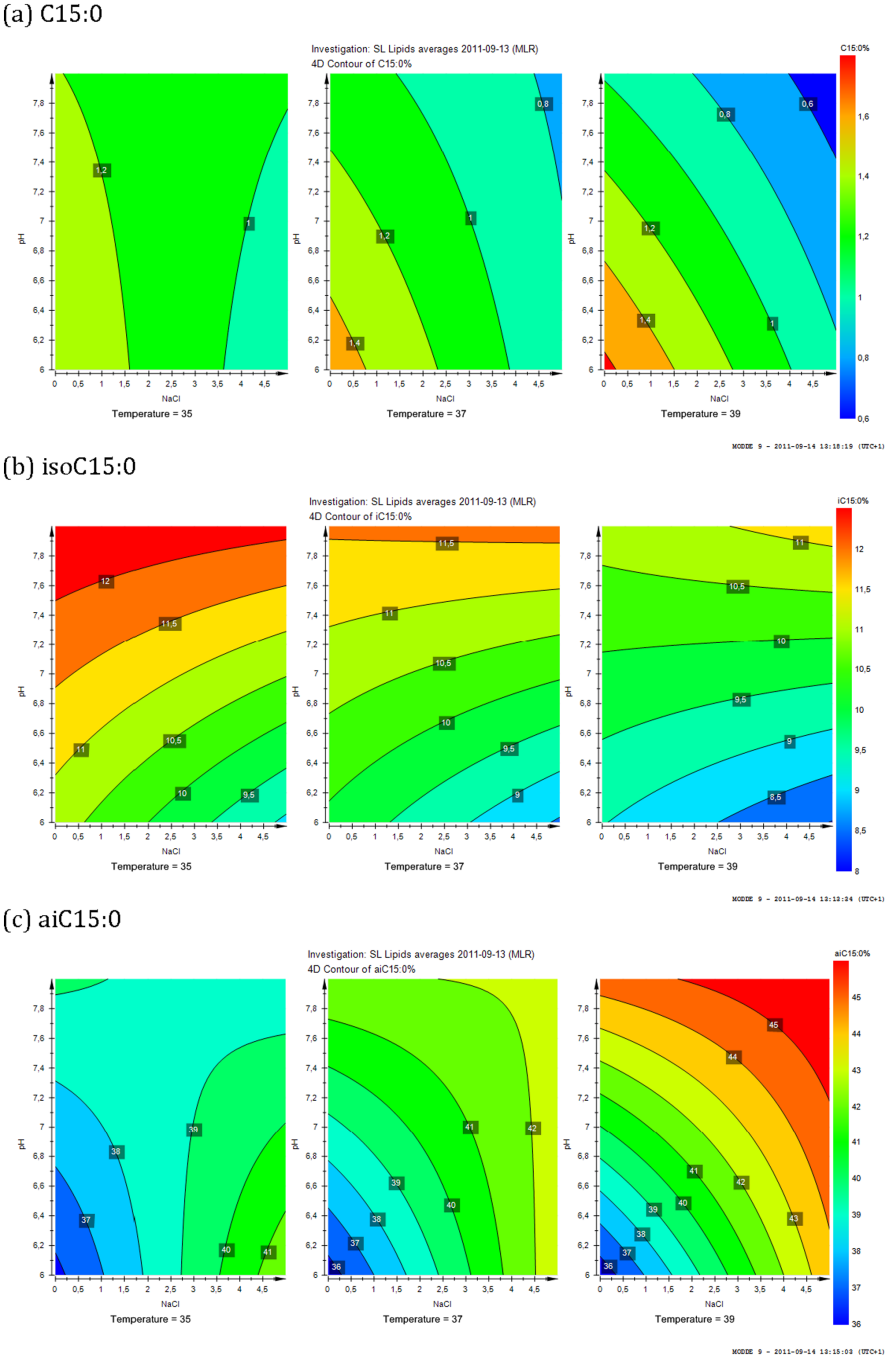
The 3D contour plots showing the relative abundances of C15∶0 (a), iC15∶0 (b) and aiC15∶0 (c) in the plasma membrane in response to the combined stresses of NaCl and pH at 35°C, 37°C and 39°C.

C15∶0 is an odd-chain fatty acid which was found to be a minor constituent (0.5–1.6%) in the plasma membrane of *S. lugdunensis* but its relative abundance was found to be significantly reduced in response to the single stresses of an additional 2.5% NaCl (centroid) as well as displaying differential responses to the treatments at 39°C (Table 2). The contour plot in Figure 5 (a) shows the combined effects of NaCl concentration and pH on the relative abundances of C15∶0 at the temperature regimes of 35°C, 37°C and 39°C. It was evident that increasing concentrations of NaCl was the major factor reducing the abundance of C15∶0 at all three temperatures. At 35°C, the pH had a minimal effect on C15∶0 levels whereas at 39°C, increasing pH contributed to the reduction of C15∶0.

The branched chain fatty acid iC15∶0 is one of the major fatty acid components present in the plasma membrane of *S. lugdunensis* (8.6–12.4%). The relative abundances of this branched chain fatty acid (BCFA) in the centroid and control samples were very similar (Table 2). The contour plot in Figure 5 (b) shows the combined effects of NaCl concentration and pH on the relative abundances of iC15∶0 at temperature regimes of 35°C, 37°C and 39°C. In contrast to C15∶0, it was evident that increasing pH was the major factor influencing the abundance of iC15∶0 at all three temperatures. At 39°C, the concentration of NaCl had a minimal effect on iC15∶0 levels, whereas at 35°C and 37°C, increasing concentrations of NaCl contributed more substantially to the reduction of iC15∶0. Decreases in temperature resulted in increased levels of iC15∶0, with conditions of lower pH and higher NaCl concentrations contributing to reduced levels of this fatty acid at all three temperature regimes.

The BCFA aiC15∶0 was the most abundant fatty acid component in the plasma membrane of *S. lugdunensis* (36–45%). Again, there was no significant difference between the relative abundances of aiC15∶0 in the control and centroid samples. The response patterns of aiC15∶0 were clearly different to those shown for C15∶0 and iC15∶0. Incubation at 39°C resulted in the highest relative abundance levels of aiC15∶0. At 39°C and 37°C, increasing pH and NaCl concentration resulted in an increase in the relative abundance of aiC15∶0 whilst at 35°C, NaCl concentration appeared to be the primary influence on the relative abundance of aiC15∶0.

## Discussion

The results from this study provided convincing evidence that *S. lugdunensis* underwent significant cellular modifications in response to subtle variations in environmental conditions. Combinations of temperature, pH and NaCl concentrations together elicited significant changes in cell morphology and membrane composition which led to measurable enhancements in antibiotic resistance and growth characteristics. Significantly increased antibiotic resistance to gentamicin was observed in cells grown under conditions of osmotic stress. The centroid samples which differed from the reference control by an additional 2.5% NaCl displayed elevated antibiotic resistance (MIC). Varying the pH and temperature parameters revealed that combinations of treatments with 5% w/w added NaCl resulted in the highest levels of MIC for gentamicin and had associated increases in generation times (Table 1). It was thus concluded that the enhanced antibiotic resistance was an inadvertent “benefit” from adaptation to the altered environmental conditions which can be defined as a process of “cross protection” [25]. Increased osmotic stress (>4.5% v/v added NaCl) or low pH (<5.0) was previously observed to increase the MIC of *S. aureus* [26] which was consistent with the results presented in this study for *S. lugdunensis*. The down regulation of synthesis of cell-wall associated proteins has been reported under conditions of higher NaCl concentrations which could impede the passage of the antibiotic through the cell wall [27] and [26]. This increased resistance was often maintained after removal of the stress providing evidence of a stable phenotype and altered metabolism in response to the stress, a feature which was able to be inherited epigenetically by successive generations.

The 5% NaCl treatment at pH 6 (35°C) displayed a higher incidence of small colony variant (SCV) formation compared with other treatments. There has been increased interest concerning the roles of *S. aureus* SCVs in persistent and relapsing infections [28] and SCVs from coagulase negative staphylococci (CoNS) have been investigated in relation to device-related bloodstream infections [29]. SCVs are a slow-growing, mostly pigmented subpopulation exhibiting substantial biochemical and physiological differences in comparison with the metabolically normal phenotype [30], [31]. When grown under optimal conditions, the generation time for SCVs is up to 9-fold longer than the corresponding wild type strains and this results in very small colonies that are frequently not visible until after 48 to 72 hours of incubation. When SCVs were exposed to antibiotics such as aminoglycosides, they exhibited a 10- to 30-fold increase in resistance compared with the parent strain [32].

When *S. lugdunensis* was grown at both 35°C and 39°C under pH 6 conditions, the addition of 5% NaCl resulted in significantly smaller cell sizes compared with the centroid samples at pH 7. Conversely, when the cells were grown at both 35°C and 39°C under pH 8 conditions, the addition of 5% NaCl resulted in significantly larger sized cells compared with the centroid. These results demonstrated how the influence of one environmental parameter (pH) could substantially alter the response of a population of cells to a second environmental parameter. This is further illustrated during growth at pH 8 where no added salt resulted in smaller cell sizes relative to the control and centroid samples at pH 7. It was evident that the narrow range of parameters tested yielded strikingly contrasting end point effects on cell size. These results suggested that significant impacts on cell structure and homeostasis were mediated by these variations in environmental conditions and collectively supported the hypothesis that *S. lugdunensis* is extremely adaptable to environmental influence.

To further investigate the adaptability of *S. lugdunensis* to the combinations of environmental stresses, the membrane fatty acid profiles were determined and analyzed by multivariate discriminate function analysis. These analyses revealed a high level of grouping of all treatment replicates into tightly packed and well resolved clusters (Figure 4). This result indicated that *S. lugdunensis* cells from each treatment regimen had characteristic and significantly different membrane fatty acid profiles as summarised in Table 2. The differences in membrane composition observed between all treatment sets supported the hypothesis that *S. lugdunensis* alters the fatty acid composition in the plasma membrane in response to subtle changes in environmental conditions as part of the adaptation mechanism. Alterations in membrane fatty acids would appear to play a significant role in the cellular response to changes in environmental conditions. A key feature of these alterations in membrane composition was the capacity for individual fatty acids to display differential response characteristics in response to the combinations of environmental conditions.

The fatty acids C15∶0, iC15∶0 and aiC15∶0 (Figure 3) were selected for evaluation of individual response characteristics as they represent a series of 15-carbon fatty acid isomers with corresponding representation as minor, mid-range or major components of the membrane fatty acid profile [33]. Each of these fatty acids displayed different response characteristics to the varying range of environmental conditions (Figure 5). This was interpreted to reflect that each of these fatty acids was under independent regulation for inclusion in the membrane dependent on the requirements for survival under each environmental regimen. This represents a new insight in regard to the dynamic nature of membrane composition in *S. lugdunensis* and suggests that these alterations may be a critical feature of the resilience and versatility of staphylococci in changing environments. Future work would focus on exploring the nature of this dynamic membrane response in other staphylococcal species and compare these with other pathogenic and non-pathogenic species.

The fatty acid aiC15∶0 has been studied extensively and its presence in the plasma membrane of *S. aureus* has been proposed as the major determinant of fluidity [34].. Increasing NaCl concentration has previously been shown to result in increased plasma membrane levels of aiC15∶0 at salt levels of 15% w/w [35]. As this fatty acid is the dominant anteiso- fatty acid present in the plasma membrane, it is possible that it was required for alkaline stress adaption in addition to salt stress. This was supported by the observation that a branched-chain alpha-keto acid dehydrogenase (BKD)-deficient mutant strain of *S. aureus* [34]. displayed significantly reduced growth kinetics when cultured in media with high pH. This BKD mutant was unable to grow at low temperatures (12°C) and when compared to the WT in fluorescence polarization, it had a much less fluid membrane [34]. The mutant exhibited half the aiC15∶0 of WT *S. aureus* suggesting that it was vital for the stress responses to increases in osmotic loads, temperature and pH [34].

The straight chain fatty acid C15∶0 is a minor membrane component of *S. lugdunensis* and an exhaustive literature search did not retrieve extensive information about its role in stress adaptation. It is a fatty acid with low abundance in many bacterial species and subsequently left out of metabolic profiling or may not be detected in analyses. *S. aureus* grown under acidic stress (pH 5) previously showed a significant decline in membrane levels of C15∶0 [36]. Although C15∶0 was sometimes only present in trace amounts, this fatty acid was responsive to many environmental stresses in the present study. It was one of the few fatty acids that were significantly altered by the sole addition of 2.5% NaCl where it decreased in concentration. It also responded variably to combined stresses: increases in acidity and temperature increased C15 levels in the membrane whereas increasing NaCl decreased its relative abundance. This was consistent with the data reported by Sado-Kamdem, [36] where *S. aureus* was cultured in a rich media (BHI) under acidic stress (pH 5).

This study provided evidence to support the hypothesis that the formation of *S. lugdunensis* phenotypes would be a continuous process in response to a constantly changing environment. These epigenetic responses would enhance cell survival whilst maintaining optimal capability for opportunistic colonization for potential host invasion. The bacteria responded to numerous combinations of subtle environmental variations resulting in cells with varying cell sizes and membrane composition that were characteristic and reproducible under each set of environmental conditions. These responses implied substantial changes in metabolic homeostasis and provided insight as to why these bacteria have evolved into such adaptive and successful pathogens.

## Conclusions

The combinations of environmental stresses including pH and osmotic pressure resulted in complex patterns of bacterial responses suggesting synergistic influences leading to changes in cell size and membrane composition with concomitant increases in gentamicin resistance. These results supported the postulate that the bacterial cell responses to each configuration of environmental conditions were optimized by underlying changes in membrane composition and cell structure to provide the most effective adaptations for survival of *S. lugdunensis*.
